# Persistent hiccups as a rare presenting symptom of empyema: a case report

**DOI:** 10.1186/s12245-024-00603-7

**Published:** 2024-02-28

**Authors:** An-Fu Lee, Hong-Wei Lee, Zui-Shen Yen

**Affiliations:** 1 Department of Emergency Medicine, Yun-Lin Branch, National Taiwan University Hospital, Douliu City, Taiwan; 2 Department of Emergency Medicine, National Taiwan University Hospital and National Taiwan University College of Medicine, No. 7, Chung-Shan S. Road, Taipei, Taiwan

**Keywords:** Hiccups, Empyema, Emergency department, Case report

## Abstract

**Background:**

Empyema is uncommon owing to antibiotic use but still affects patient health if not treated. Hiccups as the initial symptom of empyema are rare; however, empyema should be considered if a patient has persistent hiccups with unexplained fever.

**Case presentation:**

We present a case of persistent hiccups, left upper quadrant abdominal pain, and fever on day 1, and total left lung white-out and empyema on day 3. The hiccups resolved gradually after antibiotic treatment and surgical decortication.

**Conclusions:**

Clinicians should consider the possibility of empyema and conduct a chest computed tomography study if unexplained fever and persistent hiccups coexist.

## Background

Hiccups (or singultus) are involuntary diaphragmatic muscle contractions characterized by early glottic closure that terminate inspiration. Persistent hiccups without respiratory symptoms are not a typical presentation of empyema [1]. We report a rare case of empyema caused by *Streptococcus intermedius* in an immunocompetent patient with the main symptoms of persistent hiccups.

## Case presentation

**Fig. 1:**
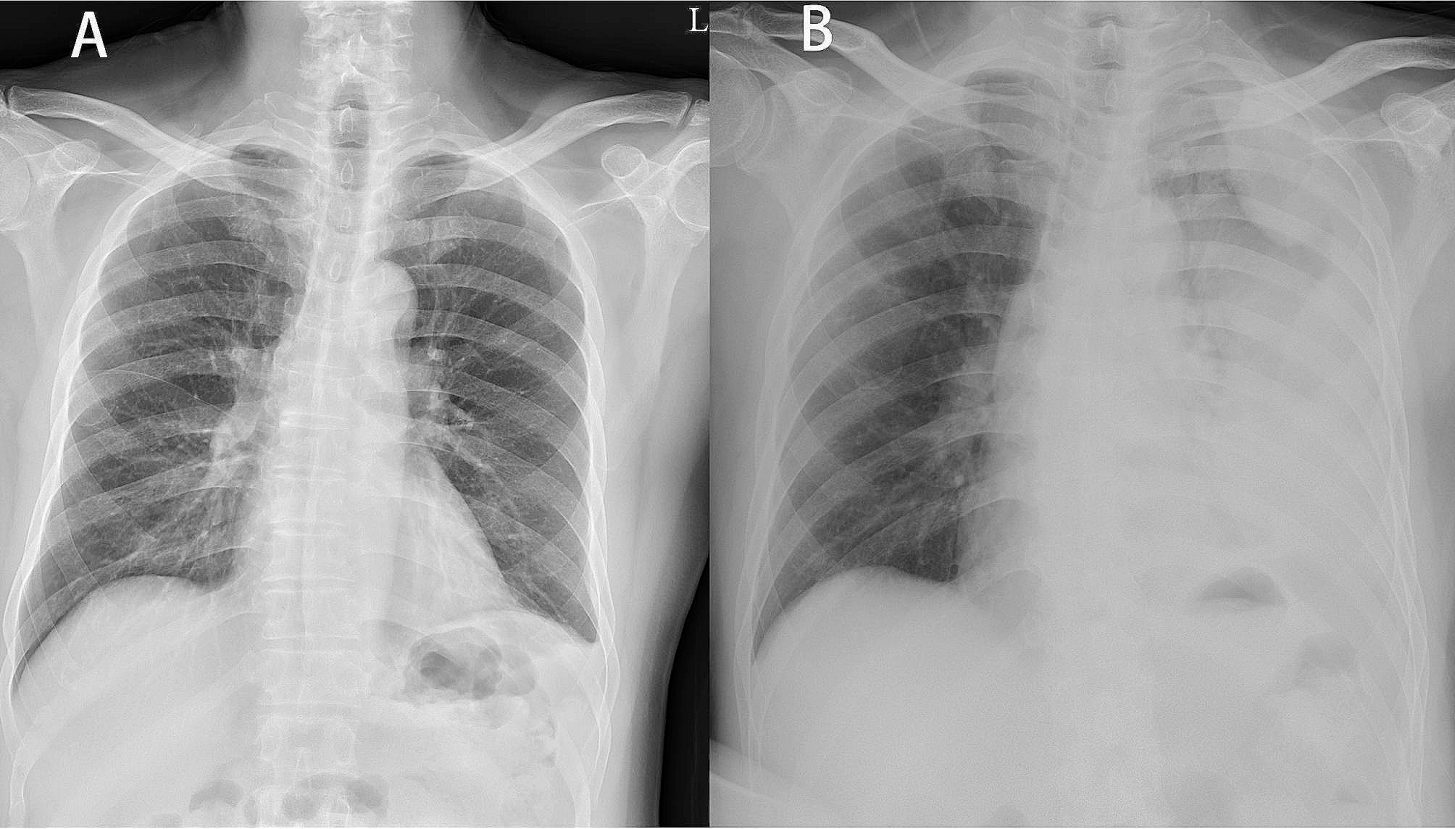
Serial chest X-rays of our patient. (**a**) Relatively normal image on Day 1. (**b**) Left lung total white-out on Day 3

A 69-year-old healthy male patient, on his first emergency department (ED) visit, presented with persistent hiccups, fever up to 38.4 °C, and mild left upper quadrant abdominal pain for 4 days. Except during sleep, his hiccup frequency was approximately several times per minute. The patient quit smoking 30 years ago after 10 years of one pack per day. He did not have URI symptoms such as cough, rhinorrhea, or sore throat. On physical examination, his vital signs included a temperature of 38.4 °C, a pulse of 107 beats/min, blood pressure of 130/73 mm Hg, respiratory rate of 18 breaths/minute, and an oxygen saturation of 95% measured by pulse oximetry while breathing ambient air. Except for mild tenderness in the left upper abdomen, the patient exhibited good oral hygiene, normal breath sounds, and all other physical examinations were normal. Fever workup revealed leukocytosis (15.34 K/µL) and minimal left pleural effusion on chest X-ray (Fig. 1a). The patient was discharged in a stable condition.

**Fig. 2:**
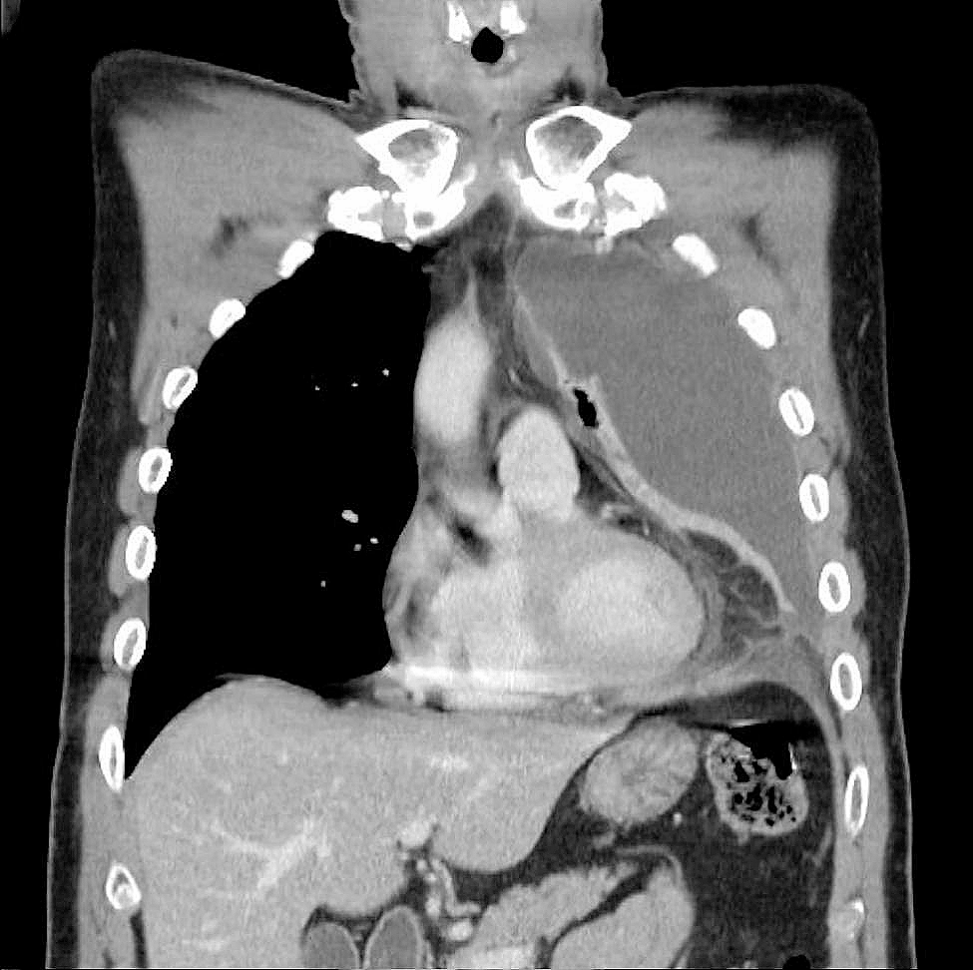
Computed tomography showed collapsed left lung with massive left pleural effusions

His hiccups persisted, and his abdominal pain progressed despite the use of analgesics. Additionally, newly developed dyspnea was noted. The patient presented to our ED 2 days after the initial visit, exhibiting vital signs: a temperature of 38.3 °C, a pulse rate of 135 beats per minute, blood pressure at 145/77 mm Hg, a respiratory rate of 24 breaths per minute, and an oxygen saturation of 94%, measured through pulse oximetry while breathing ambient air. In the physical examination, the most significant difference compared to his initial visit was a noticeable decrease in breath sounds on the left side. Laboratory data revealed leukocytosis (white blood cell count 21.39 K/µL) and lactic acidosis. Chest radiography (Fig. 1b) showed complete whitening of the left lung field. Computed tomography scans (Fig. 2) showed a collapsed left lower lung with massive left pleural effusion. Empyema was suspected.

Thoracentesis with chest tube placement was performed, and pleural fluid analysis revealed elevated levels of total nucleated cells (349/µL), total protein (4.6 g/dL), and lactate dehydrogenase (1044 U/L). Empirical ceftriaxone was administered owing to the diagnosis of empyema. The persistent hiccups and mild abdominal pain significantly improved. However, the efficacy of chest tube drainage was limited. Therefore, we changed the antibiotic from ceftriaxone to piperacillin-tazobactam. Left video-assisted thoracoscopic surgical decortication was performed.

After surgery, the patient was afebrile, and the infection profile improved with piperacillin-tazobactam. His hiccups improved and the frequency decreased to several times per day. Pleural effusion culture yielded *Streptococcus intermedius* 9 days after thoracocentesis. No bacterial growth was observed in his blood cultures by matrix assisted laser desorption ionization-time of flight mass spectrometry (MALDI-TOF MS) after 7 days. Antibiotic use was shifted to the oral form of amoxicillin-clavulanate after 14 days of intravenous antibiotic treatment. The patient was then discharged and returned to the clinic for follow-up. No significant symptoms suggestive of pulmonary infection recurrence were noted the following 6 months after discharge.

## Discussion

Many gastrointestinal and central nervous system diseases, such as esophageal cancer or brain tumors, can cause persistent hiccups by stimulating components of the hiccup reflex arc [2]. Similar to pneumonia and parapneumonic effusions, the common presentations of bacterial empyema are fever, cough, dyspnea, pleuritic chest pain, and malaise. The onset of empyema may be insidious, with patients appearing chronically ill with weight loss, anemia, and night sweats [3]. The initial presentation of persistent hiccups is very rare in bacterial empyema.

Few cases of pulmonary infection with an initial presentation of hiccups have been reported. PubMed was queried for published articles through December 31, 2023, using the search terms: “hiccup OR singultus” AND “empyema OR pneumonia”. A total of 16 case reports involving patients with respiratory infections and hiccups were identified. Excluding 10 case reports related to coronavirus disease 2019 (COVID-19), the main findings of the remaining 6 case reports [4–9] with pneumonia or empyema were summarized in Table 1. Most reported cases involved an infection in the right lower lobe. Burdette et al. suggested that inflammatory pneumonic irritation of the phrenic nerve and its pericardial branch, which are located along the superior portion of the diaphragm and right heart border, is the pathophysiological cause of persistent hiccups in cases of right middle and lower lobe pulmonary infections [8]. A similar theory has been proposed for patients infected with COVID-19, suggesting that persistent hiccups can be attributed to irritation of the vagus or phrenic nerves [10]. Most of the reported cases involved immunocompetent elderly men, all of whom survived the episodes. While hiccups are seldom mentioned as a clinical manifestation of community-acquired pneumonia, clinicians should still consider this diagnosis in patients with unexplained fever [5,6].

**Table 1:** Cases of empyema or pneumonia presenting with hiccups, excluding COVID-19 cases

	Age	Gender	Comorbidity	Infection side	Clinical presentations			Pathogen identified	Outcome
					Fever	Cough	Hiccups		
Our patient	69	Male	Smoker	LLL	**+**	**-**	**+**	*Streptococcus intermedius*	Survived
Brikman S, et al.^[[4]]^	71	Male	Hypertension	RLL	**+**	**-**	**+**	-	Survived
Konno S, et al.^[[5]]^	71	Male	Alcoholism	RLL	**+**	**-**	**+**	Legionellosis	Survived
Yamazaki Y, et al.^[[6]]^	75	Male	CVA	RLL	**-**	**-**	**+**	-	Survived
Rosenberger J, et al.^[[7]]^	44	Male	Kidney transplant	RLL	NA	NA	**+**	MRSE	Survived
Burdette SD, et al.^[[8]]^	73	Male	NA	RLL	**-**	**+**	**+**	-	Survived
Karakonstantis S, et al.^[[9]]^	79	Male	Hypertension COPD	LLL	**-**	**+**	**+**	-	Survived

Abbreviations: COPD: Chronic Obstructive Pulmonary Disease; CVA: Cerebrovascular accident; COVID-19: coronavirus disease 2019; NA: not available; MRSE: Methicillin-Resistant Staphylococcus epidermidis; RLL: Right Lower Lung; LLL: Left Lower Lung

We propose two possible explanations for the infrequent documentation of case reports involving respiratory infections presenting initially with hiccups. The first possibility is the hematogenous spreading of bacteria, allowing pathogenic microorganisms to specifically invade the alveoli near the diaphragm [11]. This invasion could then provoke an inflammatory response and stimulate the vagus or phrenic nerves, leading to initial clinical symptoms of hiccups and fever, without the presence of cough or other common pneumonia symptoms. The other explanation is that of publication bias. During the COVID-19 pandemic from 2020 to 2023, there were 10 case reports related to COVID-19 infection with hiccups. Excluding cases reporting COVID-19 infection, in the past 30 years, there have been only 6 case reports of pneumonia where hiccups were presented as a clinical manifestation. Symptoms of emerging diseases tend to attract more attention.

Our patient presented as an uncommon case of *S. intermedius* empyema, along with the atypical manifestation of persistent hiccups. *S. intermedius* is a Gram-positive coccus classified into the *Streptococcus anginosus* group (SAG). Although species in this group are normal flora in the oral cavity and gastrointestinal tract, they can sometimes cause invasive diseases, such as bacteremia, endocarditis, and abscesses in the liver and brain [12]. Respiratory infections caused by SAG bacteria tend to be observed more frequently in active smokers, male patients, and those with comorbid diseases such as liver cirrhosis and frequently involve purulent formation. Reviews by Wong et al. and Hannoodles et al. described multiple common characteristics in *S. intermedius* cases, including a mean age of 61 years and predisposing factors, such as alcohol abuse, tobacco use, periodontal disease, prior thoracic surgeries, and malignancy [13,14]. These findings align with those in our patient, who exhibited advanced age and current tobacco use. In most SAG pulmonary infections reported previously, effective treatment usually requires medical and surgical interventions. The treatment outcome was good, as previously reported in immunocompetent cases.

## Conclusions

In conclusion, the initial presentation of empyema with hiccups is rare. Clinicians should consider the possibility of empyema and conduct a chest computed tomography study if unexplained fever and persistent hiccups coexist. Antibiotics should cover *S. intermedius* if patients with a history of smoking develop empyema. Medical and surgical interventions are necessary for the treatment of *S. intermedius*-related pulmonary infections to improve prognosis.

## Data Availability

Not applicable.

## Abbreviations

ED: Emergency department
MALDI-TOF MS: Matrix assisted laser desorption ionization-time of flight mass spectrometry
SAG: *Streptococcus anginosus* group.
